# Side branch ballooning in provisional stenting of coronary bifurcation lesions: the case for a selective, imaging- and physiology-guided approach

**DOI:** 10.3389/fcvm.2026.1926839

**Published:** 2026-08-27

**Authors:** Zhangli Yuan, Yong Zhang, Dagang Huang, Xuewen Hou, Jianling Wang, Dechao Lai, Jian Xu, Yuping Xu, Gang Chen, Juan Wang

**Affiliations:** 1 Department of Cardiology, The Second People’s Hospital of Yibin, Yibin, Sichuan, China; 2 Department of Cardiology, The Fourth People’s Hospital of Yibin, Yibin, Sichuan, China; 3 Department of Radiology, The First Dongguan Affiliated Hospital, Guangdong Medical University, Dongguan, Guangdong, China

**Keywords:** bifurcation lesion, intracoronary imaging, kissing balloon technique, percutaneous coronary intervention, provisional stenting, side branch

## Abstract

Coronary bifurcation lesions account for approximately 15%–20% of all percutaneous coronary interventions (PCI) and remain among the most technically demanding scenarios in interventional cardiology. Provisional stenting—stenting the main vessel and intervening on the side branch (SB) only when necessary—is the default strategy, yet SB ballooning's role remains contested and is frequently applied reflexively. This review advances a single, evidence-based argument: SB ballooning should be deployed selectively, governed by physiology and intracoronary imaging rather than angiographic appearance, and reserved for the anatomical subsets in which it confers genuine benefit. We marshal the supporting evidence along four lines. First, mechanism: carina shift, rather than plaque shift, is a frequent and often dominant contributor to ostial SB compromise and is generally well managed without routine ballooning. Second, randomized data on strategy: in SMART-STRATEGY a conservative SB approach did not worsen long-term outcomes relative to an aggressive one and was associated with lower 3-year target-vessel failure (11.7% vs. 20.8%; a modest, single-centre, borderline-significant trial), with less periprocedural injury; EBC MAIN found no significant difference between stepwise provisional and systematic two-stent treatment in less-complex left-main bifurcations, supporting stepwise provisional stenting as default, whereas a planned two-stent strategy reduced target-lesion failure in the more complex, DEFINITION-defined subset (DEFINITION II)—so these trials should not be conflated. Third, technique optimization under imaging: the proximal optimization technique and judicious kissing-balloon inflation refine the result, and intracoronary imaging improves procedural assessment, rewiring verification, balloon sizing and optimization, while randomized trials support the clinical benefit of imaging-guided PCI in complex lesions (OCTOBER for OCT-guided bifurcation PCI; RENOVATE-COMPLEX-PCI for imaging-guided complex PCI generally)—neither trial randomized distal-cell rewiring or side-branch ballooning itself. Fourth, the device decision when the SB must be treated: the DCB-BIF randomized trial and a 2025 meta-analysis (pooled odds ratio for major adverse cardiac events 0.48) show a drug-coated balloon is a promising, evidence-supported option outperforming plain (uncoated) balloon angioplasty in appropriately selected compromised side branches, not a default for every side branch requiring dilatation. Integrating these strands, we propose a practical, lesion- and physiology-specific framework and identify principal evidence gaps future trials should address.

## 1 Introduction

### 1.1 The challenge of bifurcation PCI

Coronary artery bifurcation lesions account for approximately 15%–20% of all percutaneous coronary interventions (PCI) and consistently rank among the most technically demanding scenarios in interventional cardiology (1, 2). Their complexity arises from the interaction of fixed anatomy—two vessels of differing calibre meeting at a variable angle—with the disturbed flow and shear-stress patterns that develop at the carina and side branch (SB) ostium. The central procedural tension is the need to secure an optimal result in the main vessel (MV) while preserving the patency and function of the SB; manipulation of one branch frequently perturbs the other (3, 4). This interdependence distinguishes bifurcation PCI from the treatment of straightforward focal lesions and underlies the persistent debate over the most appropriate stenting strategy (5, 6).

### 1.2 The provisional stenting paradigm

Stenting strategies for bifurcation lesions fall broadly into single-stent (provisional) and planned two-stent approaches. In provisional stenting, the operator stents the MV across the SB ostium and escalates to SB treatment—balloon dilatation or, less commonly, stenting—only when the SB result is functionally or angiographically inadequate. Multiple randomized trials and pooled analyses have established provisional stenting as the default strategy for the majority of bifurcation lesions, owing to its procedural simplicity, shorter procedure and fluoroscopy times, and lower rates of periprocedural complications relative to systematic two-stent techniques (5, 6) (Table 1); this position is endorsed by current European Bifurcation Club (EBC) consensus (7). A planned two-stent approach remains appropriate for a defined subset—typically true bifurcations with a large, substantially diseased SB—where the likelihood of needing definitive SB treatment is high. Within the provisional framework, intracoronary imaging has become an increasingly important adjunct: optical coherence tomography (OCT)-derived plaque features, for example, are independent predictors of SB occlusion and inform pre-procedural planning (8). Lesion-modification strategies such as imaging-guided directional coronary atherectomy combined with drug-coated balloons (DCB) can, in selected cases, reduce stent burden and avoid complex stenting altogether (9), while a deliberately conservative approach to the SB has been associated with less periprocedural myocardial injury and comparable long-term outcomes (10).

**Table 1:** Bifurcation stenting strategy: provisional versus two-stent, by lesion complexity and left-main involvement.

Study (year)	Design (n)	Population & lesion type	SB size/disease criteria	Comparison/treatment trigger	Follow-up	Primary endpoint (components)	Principal result	Major limitations
Nordic + British pooled (2011/2016)	Patient-level pooled analysis of 2 RCTs	Coronary bifurcations; predominantly non-left-main	Not size-restricted	Simple (provisional) vs routine complex (two-stent)	5 y	Death, MI, TVR (MACE)	Simple strategy superior for safety and efficacy; benefit sustained at 5 y	Older DES; not powered for SB endpoints
DEFINITION (2014)	Multicentre observational (3,660)	Simple vs complex bifurcations; some left-main	Defines complexity (DEFINITION criteria)	Descriptive (validates complexity definition)	1 y	MACE	Complex 16.8% vs simple 8.9% (*p* < 0.001)	Observational; derives rather than tests a strategy
DEFINITION II (2020/2022)	RCT (653)	DEFINITION-complex bifurcations; largely non-left-main	Complex per DEFINITION (large, diseased SB)	Systematic two-stent vs provisional	1 y (3 y)	TLF (cardiac death, TV-MI, TLR)	6.1% vs 11.4% at 1 y (HR 0.52); 10.4% vs 16.0% at 3 y	Modest size; complex lesions only
DKCRUSH-V (2017)	RCT (482)	True distal left-main bifurcation	True bifurcation, diseased LCx ostium	DK crush vs provisional	1 y (3 y)	TLF	5.0% vs 10.7% (HR 0.42)	Left-main–specific; expert centres
EBC MAIN (2021)	RCT (467)	True left-main bifurcation	Investigator-defined significant SB	Stepwise provisional vs systematic two-stent	1 y (3 y)	MACE (death, MI, TLR)	1 y: 14.7% vs 17.7% (HR 0.8; *p* = 0.34; no significant difference). 3 y: primary composite 23.5% vs 29.5% (HR 0.75, 95% CI: 0.53–1.07; *p* = 0.11, ns); TLR significantly lower with provisional 8.3% vs 15.6% (HR 0.50, 95% CI: 0.29–0.86; *p* = 0.013)	Less-complex left-main; underpowered for superiority

DES, drug-eluting stent; DK, double kissing; LCx, left circumflex; MACE, major adverse cardiac events; MI, myocardial infarction; RCT, randomized controlled trial; SB, side branch; TLF, target-lesion failure (cardiac death, target-vessel MI, target-lesion revascularization); TLR, target-lesion revascularization; TV-MI, target-vessel MI; TVF, target-vessel failure; TVR, target-vessel revascularization.

### 1.3 Rationale and scope of this review

SB compromise during bifurcation PCI is not merely an angiographic nuisance: occlusion or flow-limiting stenosis of a significant SB can precipitate periprocedural myocardial infarction and, in larger branches, may carry prognostic consequences (11). SB ballooning—pre-dilatation of the SB before MV stenting, or post-stenting optimization of the jailed SB—is the principal tool for preventing and treating such compromise, and adjunctive strategies such as drug-coated balloons (DCB) and active protection with the jailed balloon technique have broadened the repertoire (12, 13) (Table 2). Yet ballooning is not without hazard, and it is frequently applied reflexively to every jailed SB irrespective of functional significance. The central thesis of this review is that this reflex is not supported by the evidence: SB ballooning should instead be selective, triggered by physiology and flow rather than angiographic appearance, guided and verified by intracoronary imaging, and reserved for lesions in which anatomy predicts genuine benefit. We develop this argument across the mechanistic, technical, and clinical-trial evidence, give particular weight to the contemporary imaging-guidance and drug-coated-balloon randomized trials, and conclude with an explicit, practical decision framework.

**Table 2:** Side-branch–specific management: ballooning, final kissing, physiology-guided intervention, active protection, and drug-coated balloons.

Study (year)	Design (n)	Population & lesion type	SB size/disease criteria	Comparison/treatment trigger	Follow-up	Primary endpoint (components)	Principal result	Major limitations
SMART-STRATEGY (2012/2016)	RCT (258)	Large bifurcation lesions; mostly non-left-main	Large SB	Conservative vs aggressive SB strategy	3 y	TVF	11.7% vs 20.8% (*p* = 0.049); equal at 1 y, benefit emerged 1–3 y	Single-centre; modest size
Nordic-Baltic III (2011)	RCT (477)	Bifurcations, single-stent main vessel; non-left-main	Not size-restricted	Final kissing-balloon vs no final kissing	8 mo (to 5 y)	Composite MACE; angiographic restenosis	Routine final kissing gave no clinical benefit after single-stent treatment	Sirolimus-stent era; not SB-size stratified
Dkcrush-VI (2015)	RCT (320)	True coronary bifurcations; non-left-main	True bifurcation (SB generally ≥2.5 mm)	FFR-guided vs angiography-guided SB intervention	1 y	Death, MI, TVR (composite)	Similar outcomes; FFR <0.80 in only ∼half of angiographically significant ostia	Modest size; open-label
J-REVERSE (2016)	IVUS-guided registry	Provisional stenting with kissing; left-main subset	Varied	Kissing-balloon inflation after provisional stenting	Short/mid-term	Angiographic (QCA)	Limited benefit in simple lesions; greater in complex bifurcations	Registry; not powered for hard endpoints
BSKT vs JWT (2019)	Comparative study	Bifurcation lesions	Varied	Balloon-stent kissing vs jailed wire	Short/long-term	Acute SB occlusion; MACE	Fewer acute SB occlusions; no difference in long-term MACE	Non-randomized
DCB-BIF (2025)	RCT (784)	SB compromised after MV stenting; left-main excluded	Compromised SB after stenting	Drug-coated vs non-compliant balloon	1 y	MACE	7.2% vs 12.5% (HR 0.56); driven by fewer TV-MI	Selected population; comparator NCB; not generalisable to left-main/very small branches
Rocchetti meta-analysis (2025)	Systematic review/meta-analysis (5 studies, 1,762)	SB treatment in bifurcation PCI	Varied	Drug-coated vs non-coated balloon	Varied	MACE (pooled)	OR: 0.48; MI OR: 0.39; no difference in TLR	Heterogeneous; includes non-randomized data
2026 meta-analysis	Systematic review/meta-analysis (6 studies, 1,982)	SB treatment in bifurcation PCI	Varied	Drug-coated vs non-coated balloon	Varied	MACE (pooled)	MI OR: 0.38 (0.19–0.76); TLR OR: 0.45 (0.23–0.87); SB late lumen loss reduced	Heterogeneous; includes observational studies and overlaps with DCB-BIF
CRABBIS (2025)	RCT (60)	Left-main/large bifurcations	Large SB	PKP (POT–kissing–final POT) vs PSP (POT–isolated SB dilatation–final POT); both arms began with POT	Procedural	Stent geometry (OCT)	Better OCT stent expansion/apposition with PKP (surrogate imaging; no clinical-outcome superiority shown)	Surrogate endpoint; small sample

FFR, fractional flow reserve; IVUS, intravascular ultrasound; MACE, major adverse cardiac events; MV, main vessel; NCB, non-compliant balloon; OCT, optical coherence tomography; OR, odds ratio; POT, proximal optimization technique; QCA, quantitative coronary angiography; SB, side branch; TLR, target-lesion revascularization; TV-MI, target-vessel MI; TVF, target-vessel failure; TVR, target-vessel revascularization.

### 1.4 Review methodology: literature search and evidence appraisal

This is a narrative review informed by a structured, non-systematic literature search. We searched PubMed/MEDLINE, Embase, and the Cochrane Central Register of Controlled Trials from database inception through 1 June 2026, with the final search performed on 1 June 2026. Search terms combined controlled vocabulary and free-text keywords for coronary bifurcation lesion, provisional stenting, side branch, kissing balloon inflation, proximal optimization technique, jailed balloon technique, drug-coated balloon, fractional flow reserve, instantaneous wave-free ratio, optical coherence tomography, and intravascular ultrasound. Reference lists of retrieved articles and the relevant European Bifurcation Club consensus documents were hand-searched. Eligible sources were English-language randomized controlled trials, meta-analyses, observational studies and registries, mechanistic and computational analyses, and consensus statements addressing side branch management during bifurcation percutaneous coronary intervention (PCI). Because this is a narrative rather than a systematic review, studies were selected and prioritised by the authors on the basis of methodological quality, directness of relevance to side branch ballooning, sample size, and recency, with disagreements resolved by discussion. In synthesising the evidence we gave greatest weight to adequately powered randomized controlled trials and their meta-analyses, followed by large registries and observational cohorts; mechanistic, imaging, and computational studies were used to explain mechanism rather than to establish clinical efficacy, and consensus documents were cited as expert opinion. We did not perform a formal risk-of-bias assessment or quantitative data pooling. This process is reported to improve transparency and to reduce the potential for selective citation; its limitations are acknowledged in the Limitations section.

## 2 Anatomical and physiological determinants of side branch vulnerability

### 2.1 Anatomical predictors of side branch compromise

**Figure 1:**
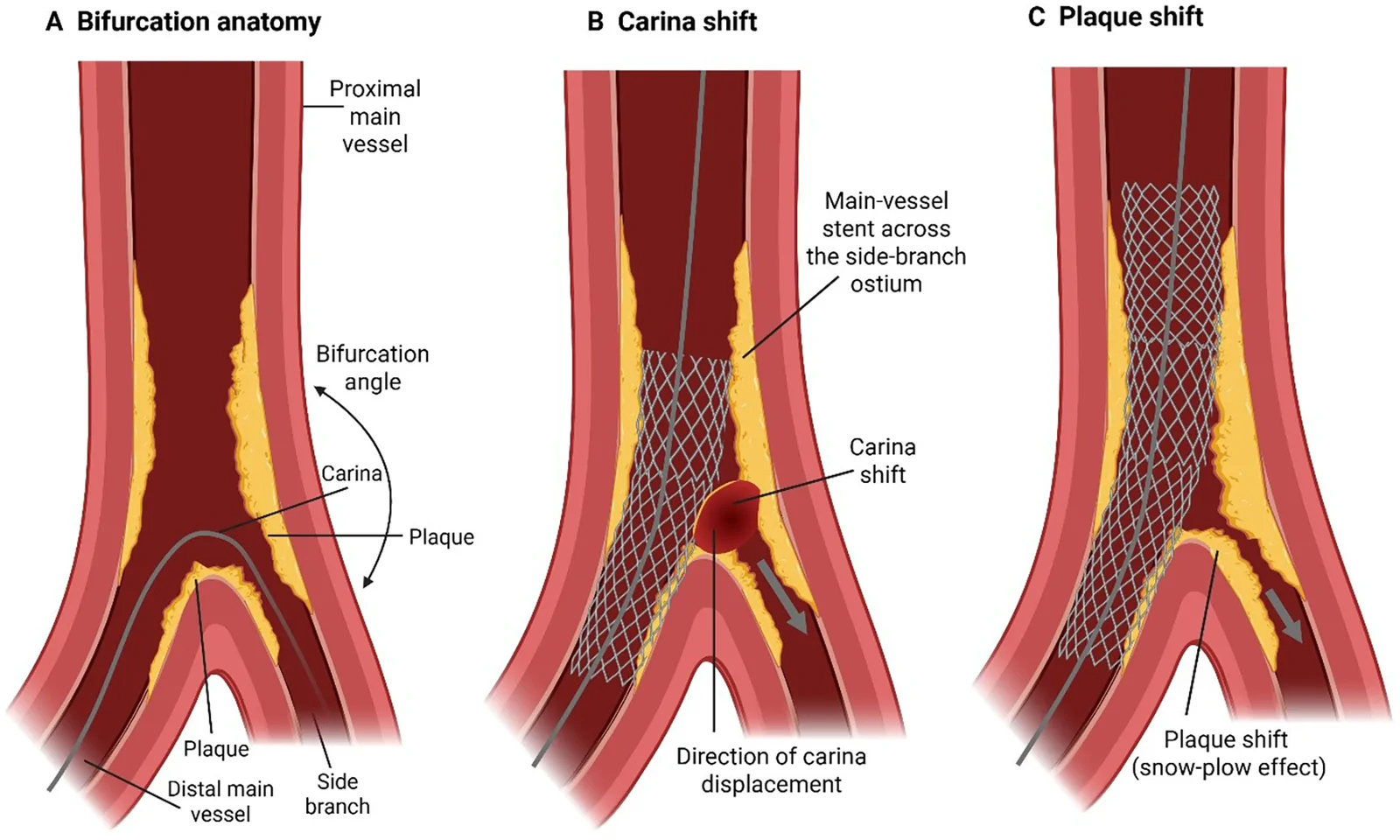
Anatomy of the coronary bifurcation and mechanisms of side branch compromise. **(A)** Longitudinal cut-away of a coronary bifurcation showing the proximal main vessel, distal main vessel, side branch, the carina (flow divider), and the bifurcation angle; atherosclerotic plaque is present in the vessel wall. **(B)** Carina shift: expansion of the main vessel stent displaces the carina toward the side branch and narrows the side-branch ostium without a change in plaque mass; the arrow indicates the direction of carina displacement. **(C)** Plaque shift (“snow-plow effect”): longitudinal redistribution of plaque from the main vessel into the side-branch ostium; the arrow indicates the direction of plaque movement. Carina shift and plaque shift frequently coexist and are the principal mechanisms of acute side-branch compromise during provisional stenting.

The anatomical configuration of a bifurcation lesion is the strongest determinant of SB outcome (Figure 1A). Bifurcation angle exerts a particularly consistent effect: a wider distal angle increases the propensity for carina shift toward the SB during MV stent expansion, raising the risk of ostial SB compromise (14, 15). Lesion length and plaque burden are likewise important, with longer and more heavily diseased segments associated with greater procedural risk. Plaque morphology adds further granularity—OCT studies have shown that lipid-rich plaque and spotty calcification at the bifurcation predict SB occlusion after provisional stenting (16, 17). Calcification compounds these risks by impeding uniform stent expansion and increasing the resistance encountered during device delivery; calcified bifurcations are correspondingly more prone to SB compromise, and dedicated calcium-modification strategies such as rotational or orbital atherectomy may be required to mitigate this hazard (17, 18). Collectively, these features reshape local flow and shear-stress distribution at the SB ostium and should be assessed—ideally with intracoronary imaging—before a stenting strategy is finalized.

### 2.2 Carina shift vs. plaque shift

Two distinct mechanisms account for most acute SB compromise, and distinguishing between them has direct therapeutic implications. Carina shift describes displacement of the flow divider toward the SB as the MV stent is expanded, narrowing the SB ostium without any change in plaque mass; plaque shift (or “snow-plowing”) describes longitudinal redistribution of plaque from the MV into the SB ostium (19, 20) (Figures 1B,C). Volumetric intravascular ultrasound (IVUS) analyses of both branches indicate that carina shift is frequently the dominant contributor to ostial SB narrowing, although the two mechanisms often coexist (19). The distinction has direct therapeutic implications, but the two mechanisms differ in their functional consequences rather than simply in whether they call for ballooning. Carina shift tends to produce marked angiographic narrowing of the ostium without an equivalent reduction in flow, so that many carina-shift ostia remain physiologically non-significant and can be managed conservatively without aggressive side-branch intervention. By contrast, extensive plaque shift and pre-existing heavy ostial disease are more likely to cause functionally important compromise and therefore more often warrant side-branch treatment or, when severe, definitive stenting. Carina shift should thus not be regarded as an automatic indication for ballooning; when the ostium is treated, kissing-balloon inflation can reshape displaced struts, but the decision to intervene should rest on flow and physiological significance rather than on angiographic appearance alone. Simulation and imaging studies further show that plaque type and SB take-off geometry modulate the magnitude of SB compromise after provisional stenting (21, 22), reinforcing the value of lesion-specific anatomical assessment.

### 2.3 Functional assessment of side branch flow

Angiographic appearance systematically overestimates the functional significance of jailed SB ostial stenoses, because of foreshortening, the eccentric morphology of the jailed ostium, and contrast streaming all distort visual assessment. Physiological interrogation therefore has a defined role. Fractional flow reserve (FFR) is the established reference standard for the haemodynamic significance of epicardial stenosis (23), and the instantaneous wave-free ratio (iFR) provides an adenosine-free alternative; below threshold values, both can identify SB lesions that may warrant further assessment or treatment—a positive physiological result identifies haemodynamic significance but does not by itself mandate ballooning, which should be weighed against branch size, subtended myocardium, flow, symptoms, dissection, and procedural risk (the evidence for FFR in jailed side branches is more developed than that for iFR) (24). The evidence that most directly informs the jailed side branch, however, comes from dedicated studies rather than from general FFR or left-main iFR series. In a prospective study of FFR in jailed side branches, most ostia that appeared severely narrowed on angiography were not functionally significant and only a minority had FFR below the ischaemic threshold, confirming that angiographic severity markedly overestimates physiological significance at the jailed ostium (25). In the randomized DKCRUSH-VI trial, FFR-guided and angiography-guided provisional side-branch intervention yielded similar one-year outcomes, and FFR was below 0.80 in only about half of the ostia that appeared significant angiographically (26). Demonstrating physiological significance, however, is not equivalent to demonstrating that ballooning improves clinical outcomes. Several caveats temper routine physiological interrogation: FFR (threshold <0.80, or the historical <0.75 used in the jailed-branch studies) is better validated in this setting than iFR, and the two indices should not be treated as fully interchangeable; measurement requires rewiring of the jailed ostium, which itself risks dissection or loss of the branch; the eccentric ostial geometry and the timing of measurement (before or after main-vessel stenting and optimization) affect the values obtained; and the additional wire, hyperaemic agent, and procedure time must be weighed against the low probability of a treatment-altering result. The pragmatic implication is that a jailed SB with preserved (TIMI 3) flow and no symptoms or ischaemic electrocardiographic changes rarely requires intervention, irrespective of its angiographic appearance—an evidence-based argument for restraint that is developed further in Section 5. This default of restraint carries two important qualifications. First, TIMI 3 flow and the absence of symptoms do not exclude a haemodynamically important stenosis in a large branch supplying substantial myocardium, and symptoms and ischaemic electrocardiographic changes may be difficult to interpret during sedated PCI; a low threshold for physiological or imaging assessment should therefore be retained for large, prognostically important branches. Second, and conversely, imaging or physiological assessment should never delay treatment when the side branch shows TIMI 0–2 or acutely occluded flow, a severe or flow-limiting dissection, persistent ischaemia, haemodynamic deterioration, or threatened loss of an important branch; these constitute urgent bail-out indications for immediate restoration of flow—by rewiring, balloon dilatation, and, if necessary, bail-out stenting—and are incorporated explicitly into the decision framework (Section 7 and the Central Illustration).

**Central Illustration:**
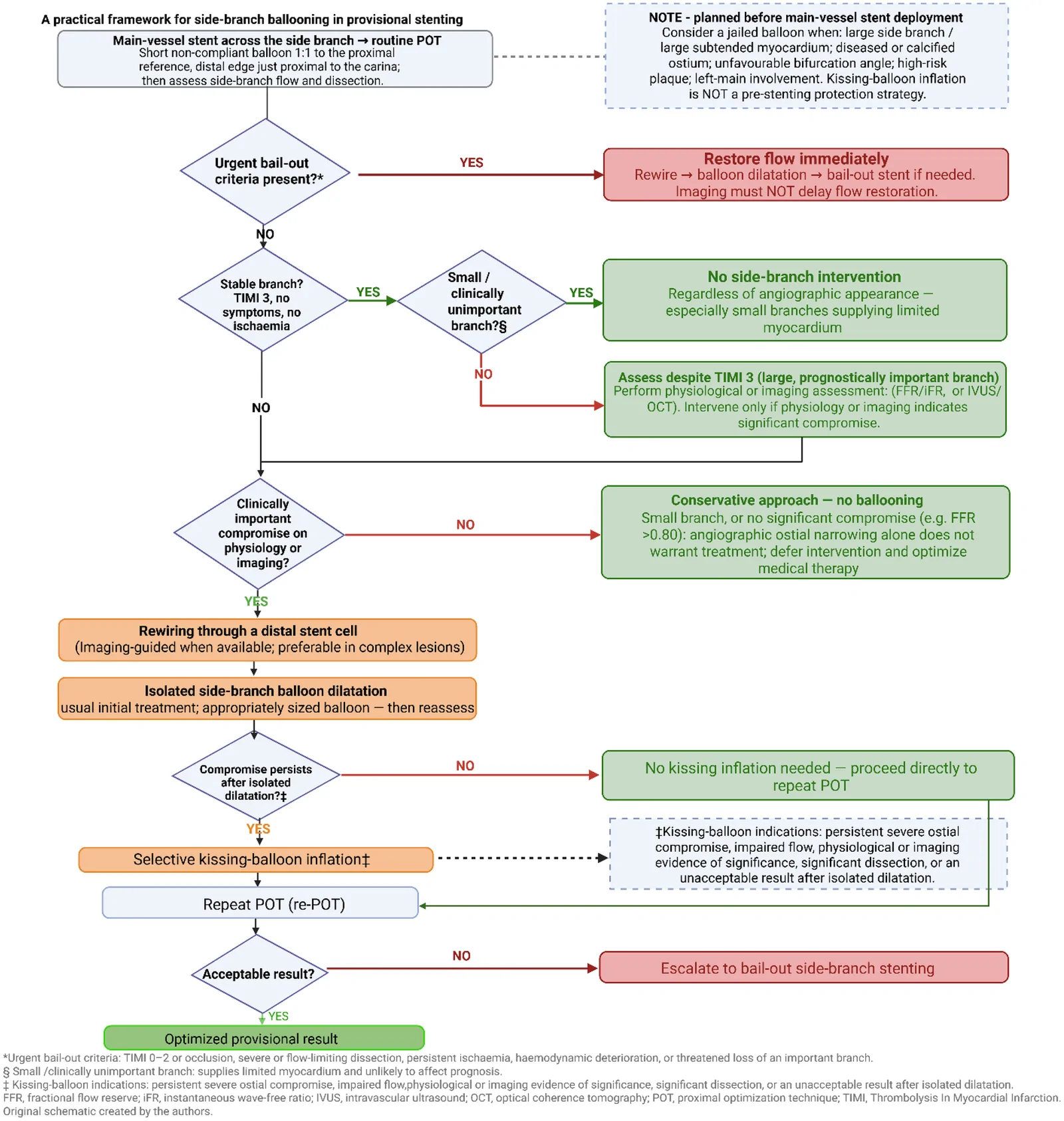
A practical framework for side branch ballooning in provisional stenting. This framework applies after main-vessel stenting across the side branch and integrates flow, physiology, imaging, and anatomy. Step 1—perform the proximal optimization technique (POT) routinely, sizing a short non-compliant balloon 1:1 to the proximal reference and positioning it just proximal to the carina, then assess side-branch flow and the ostium for dissection. Step 2 (urgent bail-out)—if the side branch shows TIMI 0–2 or occluded flow, a severe or flow-limiting dissection, persistent ischaemia, haemodynamic deterioration, or threatened loss of an important branch, restore flow immediately by rewiring, balloon dilatation, and, if needed, bail-out stenting; imaging must not delay this step. Step 3 (stable branch)—a side branch with TIMI 3 flow, no symptoms, and no ischaemia does not require intervention regardless of its angiographic appearance, particularly for small branches; for a stable but angiographically ambiguous stenosis in a large, prognostically important branch, physiological assessment (fractional flow reserve or another pressure-derived index) or intracoronary imaging may guide the decision, and clinically important compromise demonstrated by either modality supports intervention. Step 4 (when intervention is indicated)—confirm rewiring through a distal stent cell (imaging-guided where available, and preferable in complex lesions), then perform isolated side-branch balloon dilatation with an appropriately sized balloon as the usual initial treatment and reassess; reserve kissing-balloon inflation for the situations in which compromise persists (persistent severe ostial compromise, impaired flow, physiological or imaging evidence of significance, significant dissection, or an unacceptable result after isolated dilatation), and consider a drug-coated balloon for an appropriately selected compromised ostium. Step 5—perform a repeat POT (re-POT) after side-branch or kissing inflation, and escalate to bail-out side-branch stenting when an acceptable result cannot be achieved. Proactive protection with a jailed-balloon technique is planned before main-vessel stent deployment (kissing inflation is a post-stent manoeuvre and is not a pre-stenting protection strategy); the threshold for such protection is lowered by a large side branch subtending substantial myocardium, a diseased or calcified ostium, an unfavourable bifurcation angle, high-risk plaque, or left-main involvement. This is an original schematic illustration created by the authors. FFR, fractional flow reserve; POT, proximal optimization technique; TIMI, Thrombolysis In Myocardial Infarction.

### 2.4 Defining a clinically significant side branch

Because the recommendations that follow hinge on whether a side branch is worth protecting, the term “clinically significant side branch” requires an explicit, reproducible definition rather than the loose descriptors (“large,” “significant,” “important”) often used in this literature. No single threshold applies to every case, but a side branch should be regarded as clinically significant when several of the following features coincide. Anatomically, these include a reference diameter of at least 2.0–2.5 mm; a substantial subtended myocardial territory (for example, the left circumflex or a large diagonal, obtuse marginal, or dominant right-coronary branch); and a proximal location supplying a large downstream bed. Lesion-related features include ostial disease of the side branch, greater ostial stenosis severity and lesion length, a Medina classification that involves the side-branch ostium (X,X,1), and fulfilment of DEFINITION criteria for a complex bifurcation (a main criterion of side-branch diameter stenosis ≥70% with side-branch lesion length ≥10 mm for a left-main bifurcation, or ≥90% for a non-left-main bifurcation, together with minor criteria such as calcification, thrombus, main-vessel lesion length ≥25 mm, or a bifurcation angle <45° or >70°). Modifiers that raise the stakes of side-branch compromise include heavy calcification, an unfavourable (very wide or very narrow) bifurcation angle, high-risk plaque morphology at the ostium, and left-main involvement, in which the side branch typically subtends a large territory. Conversely, a small side branch (<2 mm) supplying limited myocardium, with a focal, non-flow-limiting ostial narrowing, is generally not clinically significant and rarely warrants intervention. Throughout this review, references to a “significant” or “large” side branch and to “high-risk anatomy” should be read against this composite definition, which also underpins the escalation thresholds in the practical framework (Section 7).

## 3 Techniques for Side branch ballooning

Several distinct manoeuvres are often grouped under the single heading of “side-branch ballooning,” yet they address different problems and rest on different evidence: side-branch pre-dilatation (before main-vessel stenting), jailed-balloon active protection (during stenting), post-stent dilatation of the jailed ostium, kissing-balloon inflation, drug-coated-balloon treatment of a compromised ostium, and bail-out side-branch stenting when an acceptable result cannot otherwise be achieved. Each has its own indications, mechanism, and supporting data, and they are therefore considered separately below (with drug-coated balloons discussed in Section 6.1).

### 3.1 Side branch wire protection and pre-dilatation

**Figure 2:**
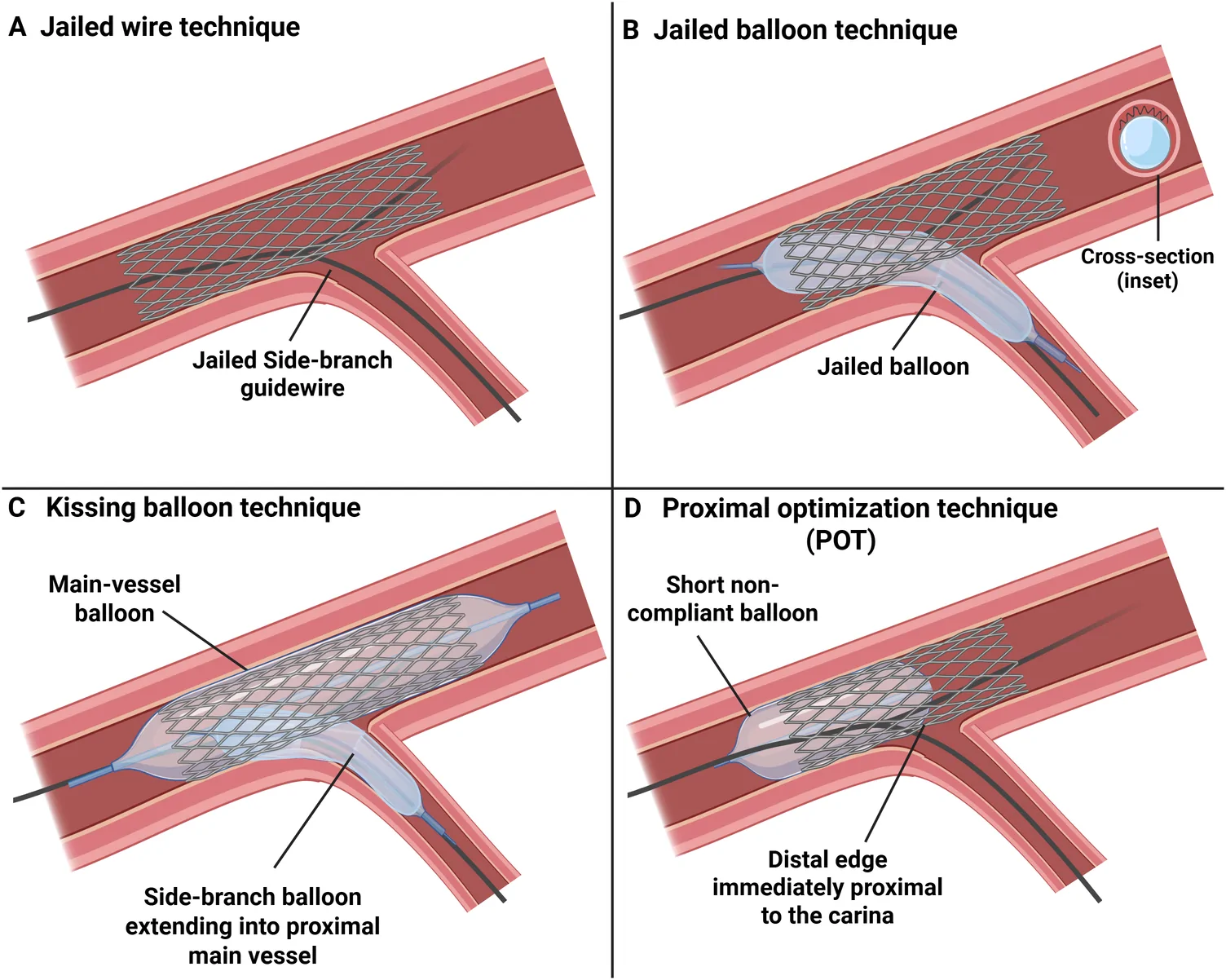
Side branch protection and ballooning techniques in provisional stenting. **(A)** Jailed wire technique: a guidewire is left in the side branch, trapped between the main vessel stent and the vessel wall, preserving access to the side branch and marking its ostium. **(B)** Jailed balloon technique: an uninflated balloon is positioned across the side-branch ostium beneath the main vessel stent so that it can be inflated immediately to restore patency if the side branch becomes compromised. The circular inset shows a cross-section taken through the stented segment, in which the deflated jailed balloon is seen compressed between the stent struts and the vessel wall. **(C)** Kissing balloon technique: simultaneous inflation of a main vessel balloon and a side branch balloon to optimize the jailed side-branch ostium and correct stent malapposition adjacent to the bifurcation. **(D)** Proximal optimization technique (POT): a short non-compliant balloon expands the proximal segment of the main vessel stent, with its distal edge positioned immediately proximal to the carina, restoring a circular stent cross-section and facilitating side-branch rewiring.

Wiring the SB before MV stenting (the “jailed wire” technique) is a near-universal safeguard in provisional stenting: it preserves access, marks the SB ostium, and may itself modestly attenuate carina shift (Figure 2A). Pre-dilatation of the SB is more selective. The principal indications are heavily calcified or high-plaque-burden bifurcations in which the SB is unlikely to be adequately treatable after MV stenting; here, pre-dilatation can facilitate stent delivery and expansion and improve subsequent SB access (27). The countervailing concern is that SB pre-dilatation may provoke plaque or carina shift, dissection, and ultimately a greater need for SB rescue (19, 20). The evidence is nuanced and of differing quality. In an observational analysis, Song et al. found that routine SB pre-dilatation before MV stenting was associated with higher rates of target-vessel failure and revascularization, suggesting that indiscriminate pre-dilatation can be counterproductive—although, being non-randomized, this finding is susceptible to confounding by indication (28); by contrast, in a randomized trial, Pan et al. reported that SB pre-dilatation did not consistently improve outcomes within a provisional T-stent strategy (29). Calcified ostia remain a recognized exception in which controlled pre-dilatation, sometimes after atherectomy, supports adequate expansion (7, 17, 30). Pre-dilatation should therefore be a deliberate, lesion-specific decision rather than a default step.

### 3.2 The kissing balloon technique

The kissing balloon technique (KBT)—simultaneous inflation of MV and SB balloons—is the classical method for optimizing the jailed SB ostium and correcting MV stent malapposition adjacent to the bifurcation (Figure 2C). By reshaping stent struts away from the SB ostium and re-apposing the proximal MV stent, KBT can improve SB access and acute angiographic results (31). Its limitations are equally well documented: simultaneous inflation imposes an elliptical, over-stretched configuration on the proximal MV segment that subsequent techniques cannot fully correct, and may induce stent deformation that has been linked to in-stent restenosis (31, 32). Refinements such as mini-KBT, in which the SB balloon is positioned to minimize overlap within the MV, aim to preserve the benefits of ostial optimization while limiting proximal distortion (31). In contemporary provisional stenting, KBT is best regarded as a selective tool—indicated chiefly when SB optimization is genuinely required—rather than a routine final step in every case. The case for restraint is supported by the randomized Nordic-Baltic Bifurcation Study III, in which routine final kissing-balloon dilatation after single-stent (main-vessel) treatment did not improve clinical outcomes compared with no final kissing, so that final kissing is not required in the majority of simple bifurcations (33). Selective kissing-balloon inflation is nonetheless reasonable when a specific problem is present: persistent severe ostial compromise of an important side branch, impaired (TIMI <3) side-branch flow, demonstrated physiological significance, a significant ostial dissection, or an unacceptable result after isolated side-branch dilatation. When the ostium is treated, a sequential approach—side-branch dilatation followed by repeat proximal optimization (re-POT)—may limit the proximal over-stretch produced by simultaneous kissing inflation while still reshaping the struts and is a reasonable alternative to routine simultaneous kissing; if simultaneous kissing is performed, it should be followed by a final POT to re-round the proximal segment. This choice should be reconciled with the CRABBIS imaging data (Section 4.2): in that small trial the kissing-containing sequence (PKP) produced better OCT-derived stent expansion and apposition than isolated side-branch dilatation (PSP), so where optimal stent geometry is the priority a kissing sequence may be preferable, whereas the sequential re-POT approach prioritises avoidance of proximal over-stretch; neither has been shown to improve clinical outcomes.

### 3.3 The proximal optimization technique

The proximal optimization technique (POT) uses a short, appropriately sized non-compliant balloon to expand the MV stent from just proximal to the carina to the proximal stent edge, matching stent dimensions to the larger proximal reference vessel (Figure 2D). POT corrects proximal malapposition, restores a circular stent cross-section, and—by opening struts toward the SB—facilitates SB rewiring through a distal cell (34). It is now widely recommended as a standard step after MV stenting and, when SB treatment is performed, as part of a POT–SB inflation–POT (“re-POT”) sequence. POT reduces global malapposition and proximal elliptical deformation, although it does not fully reverse the distortion produced by kissing-type inflations (34, 35). Balloon position is critical: finite-element analyses indicate that placement of the POT balloon immediately proximal to the carina best preserves vessel geometry and minimizes SB obstruction, whereas malpositioning can negate the technique's benefits (36, 37). Standardized, imaging-informed execution is therefore essential to realize the biomechanical advantages of POT. In practice this means sizing the POT balloon 1:1 to the proximal main-vessel reference diameter (ideally measured on intracoronary imaging), selecting a short non-compliant balloon, and positioning its distal edge immediately proximal to the carina. These details matter because the technique is not benign when performed incorrectly: an oversized balloon or a balloon positioned distal to the carina can worsen vessel geometry, provoke carina shift, and increase—rather than relieve—side-branch compromise. A repeat POT should also be performed after any side-branch intervention (the POT–side-branch inflation–POT, or “re-POT,” sequence) to re-round the proximal stent and correct the deformation induced by side-branch or kissing inflation.

### 3.4 The jailed balloon technique and other active protection strategies

When the SB is at high risk of acute occlusion, active protection extends beyond a jailed wire. In the jailed balloon technique (JBT), an uninflated or partially inflated balloon is positioned across the SB ostium during MV stent deployment; if the SB is compromised, the balloon can be inflated immediately to restore patency without the need to re-cross a jailed stent (Figure 2B). Subgroup data from a randomized comparison indicate that JBT reduces acute SB occlusion relative to the jailed wire technique in lesions at high predicted risk (11). Related balloon-embedded and balloon-stent kissing approaches similarly aim to maintain a channel to the SB during MV stenting (13, 38); the 17th EBC consensus document is dedicated specifically to techniques for preserving and restoring SB access during stepwise provisional stenting (39). These techniques improve acute SB outcomes but add procedural complexity and have not been shown to alter long-term clinical events, underscoring that their value lies chiefly in high-risk anatomy.

### 3.5 Intracoronary imaging guidance

Intracoronary imaging is integral to contemporary SB management. OCT, with its high spatial resolution, is particularly suited to characterizing the jailed SB ostium, verifying stent-strut position relative to the ostium, and confirming successful rewiring through a distal cell before SB ballooning (40). Three-dimensional OCT reconstruction has demonstrated that the jailed SB ostium frequently has an eccentric morphology that influences rewiring and ballooning strategy (40). IVUS, with its greater depth of penetration, is well suited to assessing proximal MV reference dimensions, plaque burden, and the adequacy of stent and balloon expansion, and to guiding POT balloon sizing. The two modalities are complementary rather than interchangeable. OCT, with its higher resolution, is particularly useful for defining stent-strut configuration at the ostium and for confirming rewiring through the distal cell, aided by three-dimensional reconstruction; IVUS, with its greater depth of penetration, is preferred for vessel and reference-lumen sizing, quantifying plaque burden, assessing stent expansion, and evaluating the left main and aorto-ostial segments. Combined or sequential use can therefore improve the accuracy of anatomical assessment in complex bifurcations (Table 3). These advantages are offset by practical limitations—additional cost and capital equipment, variable availability, added procedure time, and, for OCT, the need for extra contrast (a consideration in renal impairment)—which constrain routine use without diminishing the value of imaging where it is available. Imaging also mitigates the interpretive pitfalls of angiography alone—foreshortening, vessel overlap, and contrast streaming—that lead to both over- and under-estimation of SB compromise; systematic, real-time image acquisition and interpretation reduce these errors and support more consistent decision-making.

**Table 3:** Comparison of optical coherence tomography (OCT) and intravascular ultrasound (IVUS) for assessment and treatment of coronary bifurcation lesions.

Feature	Optical coherence tomography (OCT)	Intravascular ultrasound (IVUS)
Spatial resolution	High (∼10–20 µm), superior for fine structural detail	Lower (∼100–150 µm)
Penetration depth and contrast	Shallow (∼1–2 mm); requires a contrast or dextran flush	Deep (visualises the full vessel-wall thickness); no contrast required
Particular strengths at the bifurcation	Defining stent-strut configuration at the ostium; confirming rewiring through a distal cell; three-dimensional reconstruction of the jailed ostium	Reference-vessel and lumen sizing; plaque burden; stent expansion; assessment of the left main and aorto-ostial segments
Main practical limitations	Limited penetration; additional contrast (caution in renal impairment); less suited to large ostial left main vessels	Lower resolution; less detailed depiction of struts and of distal-cell rewiring
Preferred role in side branch intervention	Verifying distal-cell rewiring position and the ostial ballooning result	Sizing POT and kissing balloons and assessing the left main

IVUS, intravascular ultrasound; OCT, optical coherence tomography; POT, proximal optimization technique.

The clinical value of routine imaging guidance in this setting is now supported by randomized evidence. The RENOVATE-COMPLEX-PCI trial randomized 1,639 patients with complex coronary lesions (2:1 to imaging-guided vs. angiography-guided PCI) and found that intravascular imaging guidance reduced target-vessel failure—a composite of cardiac death, target-vessel myocardial infarction, and clinically driven target-vessel revascularization—compared with angiography alone (41). The bifurcation-specific OCTOBER trial randomized 1,201 patients with complex bifurcation lesions to OCT-guided or angiography-guided PCI and reported a lower 2-year rate of MACE with OCT guidance (10.1% vs. 14.1%) (42), albeit at the cost of longer procedure times and greater contrast use. The benefit of imaging guidance proved durable: at a median 5.3 years, RENOVATE-COMPLEX-PCI maintained a lower rate of target-vessel failure with intravascular imaging (10.5% vs. 14.9%; hazard ratio 0.68, 95% CI: 0.51–0.91) (43). Together these trials support a structured, imaging-guided approach to bifurcation PCI, including verification of SB rewiring position and of the SB ballooning result. The strength of this evidence should not be over-stated, however. OCTOBER provides bifurcation-specific data, whereas RENOVATE-COMPLEX-PCI enrolled a broader complex-PCI population and did not randomize patients according to imaging-guided side-branch ballooning; neither trial demonstrates that imaging must govern every side-branch intervention. These results therefore endorse an imaging-informed strategy for complex bifurcations rather than mandating imaging before every side-branch manoeuvre, and imaging should never delay restoration of flow in an acutely compromised branch (Table 4).

**Table 4:** Intracoronary imaging guidance in complex and bifurcation PCI.

Study (year)	Design (n)	Population & lesion type	SB size/disease criteria	Comparison/treatment trigger	Follow-up	Primary endpoint (components)	Principal result	Major limitations
RENOVATE-COMPLEX-PCI (2023)	RCT (1,639)	Complex lesions incl. bifurcations; NOT SB-specific	Broad (not SB-defined)	Imaging-guided vs angiography-guided PCI	median 2.1 y (5.3 y)	TVF (cardiac death, TV-MI, TVR)	2.1 y: 7.7% vs 12.3% (HR 0.64). 5.3 y: 10.5% vs 14.9% (HR 0.68, 95% CI: 0.51–0.91)	Broad population; not randomized by SB ballooning
OCTOBER (2023)	RCT (1,201)	Complex bifurcations (18% left-main)	Bifurcation-specific	OCT-guided vs angiography-guided PCI	2 y	MACE (cardiac death, TV-MI, TLR)	10.1% vs 14.1% (HR 0.70)	Longer procedure time and greater contrast use

MACE, major adverse cardiac events; OCT, optical coherence tomography; PCI, percutaneous coronary intervention; SB, side branch; TLR, target-lesion revascularization; TV-MI, target-vessel myocardial infarction; TVF, target-vessel failure; TVR, target-vessel revascularization. Numbers are reported as intervention versus comparator.

## 4 Clinical evidence for side branch ballooning

### 4.1 Landmark trials and registries

**Figure 3:**
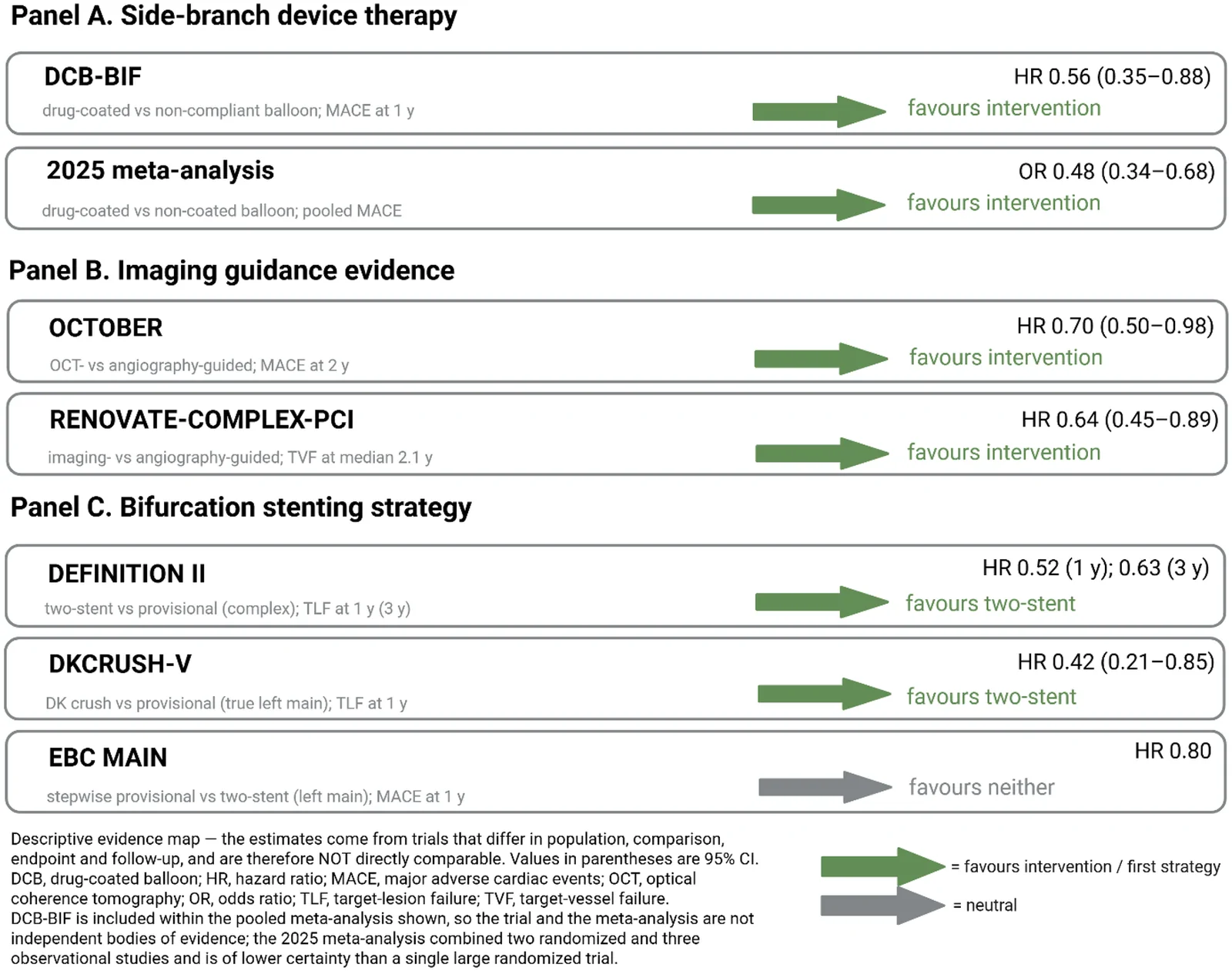
Evidence map of selected trial and pooled effect estimates relevant to side-branch management, grouped into three clinically coherent panels so that non-comparable interventions are not displayed together. Each value is the point estimate reported in the source publication. Because the trials differ in population, comparison, endpoint, and follow-up, the estimates are not directly comparable and are presented descriptively rather than as a forest plot. **(A)** Side-branch devices (favouring the drug-coated balloon): DCB-BIF (drug-coated versus non-compliant balloon for a compromised side branch; MACE at 1 year) and the pooled drug-coated-balloon meta-analysis (2025). **(B)** Imaging guidance (favouring imaging): OCTOBER (OCT- versus angiography-guided PCI; MACE at 2 years) and RENOVATE-COMPLEX-PCI (imaging- versus angiography-guided complex PCI; target-vessel failure at median 2.1 years). **(C)** Bifurcation stenting strategy: DEFINITION II and DKCRUSH-V favoured a planned two-stent strategy in DEFINITION-complex and true left-main bifurcations respectively, whereas EBC MAIN showed no statistically significant difference between stepwise provisional and systematic two-stent treatment (favours neither). All estimates are hazard ratios except the meta-analyses (odds ratio); a value below 1.0 favours the first-named strategy. DCB-BIF is included within the pooled meta-analysis shown, so the trial and the meta-analysis are not independent bodies of evidence; the 2025 meta-analysis combined two randomized and three observational studies and is of lower certainty than a single large randomized trial. This is a descriptive evidence map, not a new pooled meta-analysis, and individual estimates should not be compared against one another visually. DCB, drug-coated balloon; LM, left main; MACE, major adverse cardiac events; NC, non-compliant; OCT, optical coherence tomography; TLF, target-lesion failure; TVF, target-vessel failure.

The evidence base for bifurcation stenting strategy is substantial, but evidence specific to SB ballooning is more limited and heterogeneous (Tables 1, 2). The J-REVERSE registry evaluated IVUS-guided kissing-balloon inflation after provisional stenting and found limited incremental benefit in simple lesions but greater apparent efficacy in complex bifurcations (44). This pattern—benefit concentrated in complexity—recurs throughout the literature. The patient-level pooled analysis of the Nordic Bifurcation Study and the British Bifurcation Coronary Study established the superiority of a simple (provisional) strategy over routine complex stenting, sustained at 5-year follow-up (45, 46). Throughout, the composite endpoints used by individual trials are not interchangeable and their components differ: major adverse cardiac events (MACE) generally denote a composite of death, myocardial infarction, and revascularization; target-lesion failure (TLF) comprises cardiac death, target-vessel myocardial infarction, and target-lesion revascularization; and target-vessel failure (TVF) comprises cardiac death, target-vessel myocardial infarction, and target-vessel revascularization. The exact definitions in each cited trial are as reported in the source publications. The DEFINITION study provided criteria to distinguish simple from complex lesions and showed that complex lesions carry substantially higher rates of major adverse cardiac events (MACE)—a 1-year MACE rate of 16.8% vs. 8.9% in simple lesions (*p* < 0.001) (47). The corollary question—whether complex lesions therefore warrant an upfront two-stent strategy—has been tested directly. In DEFINITION II (653 patients with complex bifurcations), a systematic two-stent approach reduced 1-year target-lesion failure relative to provisional stenting [6.1% vs. 11.4%; hazard ratio (HR) 0.52, 95% CI: 0.30–0.90], a difference that persisted but did not widen at 3 years (10.4% vs. 16.0%; HR: 0.63, 95% CI: 0.41–0.97) (48). In the left main, DKCRUSH-V found double-kissing crush superior to provisional stenting for true distal left-main bifurcations (1-year target-lesion failure 5.0% vs. 10.7%; HR 0.42) (49), whereas EBC MAIN, which was not designed as a formal non-inferiority trial, demonstrated no statistically significant difference between stepwise provisional and systematic two-stent treatment (1-year MACE 14.7% vs. 17.7%; HR: 0.8, *p* = 0.34), with a numerical trend favouring the provisional strategy, and supported stepwise provisional stenting as the default approach (50). At 3-year follow-up, EBC MAIN continued to show no statistically significant difference in the primary composite of death, myocardial infarction and target-lesion revascularization between the strategies (23.5% vs. 29.5%; hazard ratio: 0.75, 95% CI: 0.53–1.07; *p* = 0.11), whereas target-lesion revascularization was significantly lower with the stepwise provisional strategy (8.3% vs. 15.6%; hazard ratio: 0.50, 95% CI: 0.29–0.86; *p* = 0.013) (51). The practical reconciliation, reflected in the most recent European Bifurcation Club consensus, is that a stepwise provisional strategy should remain the default for the majority of bifurcations, with an upfront two-stent approach reserved for genuinely complex anatomy and large, heavily diseased side branches (52). A recurring limitation across this body of work is constrained sample size and follow-up for SB-specific endpoints, which weakens inferences about SB ballooning in particular. The selected trial and pooled effect estimates discussed in this section and in Section 5 are summarized graphically in Figure 3.

### 4.2 Outcomes with vs. without side branch ballooning

Direct comparisons of provisional stenting with and without SB ballooning reveal a consistent dissociation between acute and long-term results: SB ballooning reliably improves the immediate angiographic and physiological SB result, but this acute gain does not translate consistently into a reduction in long-term clinical events. In the 3-year analysis of SMART-STRATEGY (258 patients with large bifurcation lesions), a conservative SB strategy reduced target-vessel failure relative to an aggressive strategy (11.7% vs. 20.8%; *p* = 0.049); the strategies were equivalent at 1 year (9.4% vs. 9.2%), with the advantage of restraint emerging between years 1 and 3 (2.6% vs. 12.7%; *p* = 0.004), alongside less procedure-related myocardial injury (10, 53). Technique comparisons point the same way: the balloon-stent kissing technique and the jailed wire technique differ in acute SB occlusion but not in long-term MACE (38). Where the SB result is suboptimal, however, the choice of how to optimize it matters. The CRABBIS trial, a small (60-patient), single-centre randomized OCT study in which both arms began with proximal optimization, compared POT–kissing-balloon inflation–final POT (PKP) with POT–isolated side-branch dilatation–final POT (PSP); the PKP sequence achieved better OCT-derived stent expansion and apposition, a surrogate-imaging finding that does not by itself demonstrate a clinical-outcome benefit (54). The overarching implication is that the benefit of SB ballooning is largely procedural and geometry-dependent, and that routine, indiscriminate SB ballooning is unlikely to improve prognosis in unselected lesions.

### 4.3 Lesion-specific considerations

Lesion type modulates both strategy and outcome. Left main coronary artery (LMCA) bifurcations are a distinct category: given their large myocardial territory, SB (typically left circumflex) compromise carries greater consequence, yet observational data still favour a one-stent over a two-stent strategy for many LMCA bifurcations when feasible (55). Bifurcation angle influences procedural behaviour—COBIS registry data link a lower bifurcation angle to a greater need for interventional SB procedures—although its independent effect on long-term MACE is less clear (56). SB calibre and the extent of ostial disease remain the most actionable determinants: a large SB subtending substantial myocardium, or a heavily diseased ostium, shifts the balance toward more proactive SB treatment, whereas a small SB with a focal, non-flow-limiting ostial narrowing rarely warrants intervention.

## 5 Controversies and unresolved questions

### 5.1 Is side branch ballooning always necessary?

The most fundamental controversy is whether the SB requires intervention at all when it is asymptomatic, has preserved (TIMI 3) flow, and is not functionally significant. Several lines of evidence support restraint. SB ballooning can provoke dissection and plaque shift and, when performed routinely, may increase MV-related events—Song et al. linked routine SB pre-dilatation to higher target-vessel failure and revascularization (28). SMART-STRATEGY showed that a conservative strategy did not worsen long-term outcomes relative to an aggressive one—and was associated with lower 3-year target-vessel failure (a modest, single-centre trial of borderline significance)—with less myocardial injury (10, 53). When the SB genuinely must be treated, the device decision is now supported by randomized and pooled data: the DCB-BIF trial (784 patients) reduced 1-year MACE with a drug-coated balloon vs. non-compliant balloon angioplasty (7.2% vs. 12.5%; HR: 0.56, 95% CI: 0.35–0.88; *p* = 0.013), driven by fewer target-vessel myocardial infarctions (57), and a 2025 systematic review and meta-analysis of five studies (1,762 patients) found that DCB treatment of the SB lowered MACE (pooled odds ratio: 0.48, 95% CI: 0.34–0.68; *I*^2^ = 0%) and myocardial infarction (odds ratio: 0.39, 95% CI: 0.25–0.62) compared with non-coated balloons, with no significant difference in target-lesion revascularization (58). A more recent (2026) systematic review and meta-analysis of six studies (1,982 patients) similarly reported lower myocardial infarction with side-branch drug-coated-balloon treatment (odds ratio: 0.38, 95% CI: 0.19–0.76) (59); both meta-analyses, however, incorporate the DCB-BIF trial and include predominantly observational studies, so they are consistent with—rather than independent of—that trial and carry lower certainty than a single large randomized trial. The weight of evidence therefore favours a selective, physiology- and flow-guided approach to whether the SB is treated, and—for an appropriately prepared, compromised side branch resembling the DCB-BIF population—a drug-coated balloon over plain angioplasty when it is treated, recognising that this evidence does not extend to left-main lesions, severe calcification, very small branches, or other drug-coated-balloon platforms.

### 5.2 Optimal timing of side branch intervention

When SB treatment is undertaken, its timing relative to MV stenting remains debated. Pre-dilatation before MV stenting may improve SB access and reduce the need for difficult rescue manoeuvres, but it risks plaque or carina shift and may commit the operator to additional intervention (29). Post-stenting optimization—rewiring through a distal cell followed by SB ballooning and POT—preserves the provisional philosophy of treating the SB only when its result proves inadequate, but it depends on successful rewiring of a jailed ostium. Timing also interacts with vascular healing: experimental and computational work shows that the mode and sequence of stent and balloon expansion influence wall stress, vascular injury, and subsequent endothelialization (60), and that healing responses differ across stent platforms (61). These data argue for an individualized sequence informed by lesion anatomy and imaging rather than a fixed protocol.

### 5.3 Long-term safety and evidence gaps

Long-term safety data specific to SB ballooning are notably sparse. Most trials in this area are powered for acute or intermediate-term endpoints, leaving the extended consequences of SB ballooning—particularly its effect on SB ostial restenosis, the durability of SB patency, and any interaction with MV stent failure—incompletely characterized. Heterogeneity in lesion definitions, SB-treatment thresholds, technique execution, and endpoint adjudication further limits cross-study comparison and meta-analysis. The field would benefit from adequately powered, imaging-adjudicated randomized trials with multi-year follow-up and standardized SB endpoints; until these are available, recommendations regarding SB ballooning rest on a foundation of predominantly short-term evidence.

## 6 Emerging technologies and future directions

### 6.1 Drug-coated balloons in side branch intervention

DCBs deliver antiproliferative drug to the vessel wall without leaving a permanent implant, an attractive proposition at the bifurcation, where additional metal in the SB increases the risk of malapposition, restenosis, and thrombosis (62, 63). Established coronary indications for DCB include in-stent restenosis and *de novo* disease in small vessels, and the absence of an implant can permit shorter dual antiplatelet therapy in patients at high bleeding risk (a consideration that applies chiefly to stand-alone drug-coated-balloon use; in the provisional strategy discussed here a drug-eluting stent remains implanted in the main vessel, so antiplatelet duration is ordinarily governed by the main-vessel stent, clinical presentation, and bleeding risk rather than by drug-coated-balloon use in the side branch) (62–64). In the bifurcation SB specifically, the randomized DCB-BIF trial provides the strongest evidence to date that DCB treatment improved one-year outcomes relative to non-compliant balloon angioplasty, albeit in a selected population with a side branch compromised after main-vessel stenting (57). Important limitations remain: variability and incompleteness of drug transfer, the need for adequate lesion preparation, and a still-limited body of dedicated randomized data. In particular, DCB-BIF compared a drug-coated balloon with non-compliant balloon angioplasty (not with a drug-eluting stent); its reduction in major adverse cardiac events was driven largely by fewer target-vessel myocardial infarctions rather than by a clearly demonstrated reduction in side-branch revascularization; and its results should not be generalized to left-main lesions, severe calcification, very small branches, or other drug-coated balloon platforms. A drug-coated balloon is therefore best regarded as a promising, evidence-supported option for appropriately selected compromised side branches rather than a default whenever any side branch requires treatment. The optimal role of DCB in SB intervention, including direct comparison with drug-eluting stents in this setting—requires further definition (62–64).

### 6.2 Bioresorbable scaffolds for side branches

Bioresorbable scaffolds aim to provide transient mechanical support and then resorb, potentially avoiding the long-term sequelae of permanent metallic caging of the SB ostium. Realizing this promise depends on matching scaffold mechanical performance and degradation kinetics to the timeline of vascular healing: resorption that is too rapid risks premature loss of support, while degradation products and their local effects must be biocompatible (65). Magnesium-, zinc-, and iron-based platforms are under investigation, with zinc alloys of particular interest for their intermediate degradation rate and tunable biocompatibility (66), and metal-polymer composite designs proposed to modulate the degradation of iron-based scaffolds (67). First-generation polymeric scaffolds were limited by strut thickness and adverse late events, and bifurcation-specific bioresorbable technology for the side branch remains investigational with no dedicated clinical evidence; it is therefore noted here only briefly.

### 6.3 Computational modeling and artificial intelligence

Patient-specific computational modeling allows stenting strategies to be simulated before the procedure. Finite-element and computational fluid-dynamics analyses of bifurcation stenting are feasible and can predict the mechanical and haemodynamic consequences of alternative approaches—including POT balloon position and kissing-inflation strategy—supporting individualized pre-procedural planning (68). Expert biomechanical-modeling reviews have outlined how such analyses can be translated into technique optimization to reduce malapposition, restenosis, and thrombosis (69). Artificial intelligence is beginning to augment this work, with applications in automated angiographic and intracoronary-imaging analysis and in pre-procedural planning. The demonstrated benefit of structured, protocolized imaging guidance in randomized trials such as OCTOBER (42) illustrates the value of standardized, algorithmic decision support. Artificial-intelligence tools that might build on this foundation remain unready for routine use and require prospective validation and are noted here only briefly.

### 6.4 Special scenario: the inverted provisional strategy

One special scenario deserves brief mention. Isolated ostial left-circumflex disease without left-anterior-descending involvement (Medina 0,0,1) carries higher follow-up event rates and is challenging at the circumflex ostium, and the optimal side-branch strategy is debated. For such lesions the inverted provisional technique—stenting the branch conventionally treated as the side branch—has shown high procedural success and favourable outcomes in small observational series, with frequent use of intravascular ultrasound and proximal optimization (70). The inverted provisional strategy is therefore a useful option for these lesions, though it remains peripheral to the central question of side-branch ballooning and rests on observational evidence that requires prospective confirmation.

### 6.5 Priorities more directly relevant to side branch ballooning

Several developments bear more directly on side-branch management than bioresorbable scaffolds or artificial intelligence and deserve emphasis. Angiography-derived physiology (for example, computational fractional flow reserve and related indices) may allow the functional significance of a jailed ostium to be estimated without a pressure wire, potentially lowering the practical barriers to physiology-guided decisions—although it requires dedicated validation in the specific geometry of the jailed side branch. Dedicated bifurcation devices and drug-coated-balloon platforms tailored to the side-branch ostium warrant continued evaluation against the provisional standard. Beyond technology, procedural standardization—consistent use of POT, distal-cell rewiring, and re-POT, verified by imaging—is likely to yield more reproducible results than any single device. Finally, the outcomes that matter most for this question, side-branch ostial restenosis and the long-term patency of the treated branch, remain poorly characterized; adequately powered trials with standardized, side-branch-specific endpoints and multi-year follow-up are the key unmet need.

## 7 Toward a practical framework for side branch ballooning

Synthesizing the foregoing evidence, SB ballooning in provisional stenting is best approached as a selective, lesion- and patient-specific decision rather than a routine step (Central Illustration; Table 5). Several principles emerge. First, the default after MV stenting in a true bifurcation should be POT, followed by SB intervention only if the SB result is inadequate. Second, the threshold for SB ballooning should be functional and flow-based: a jailed SB with TIMI 3 flow, no symptoms, and no ischaemic electrocardiographic change generally does not require treatment, whatever its angiographic ostial appearance. Third, anatomy should drive escalation—a large SB subtending substantial myocardium, a heavily diseased or calcified ostium, an unfavourable bifurcation angle, or high-risk plaque morphology all lower the threshold for proactive protection with a jailed-balloon technique—planned before main-vessel stent deployment, since kissing inflation is a post-stent manoeuvre rather than a pre-stenting protection strategy—or for definitive SB treatment. Fourth, when the SB is treated, intracoronary imaging should, where available, guide rewiring, balloon sizing, and verification of the result, and a re-POT sequence should follow kissing inflation. Fifth, when SB compromise requires treatment, a DCB is an evidence-supported alternative to plain balloon angioplasty. This framework prioritizes simplicity and restraint for the majority of lesions while reserving more intensive SB intervention for the anatomical subsets in which the evidence indicates genuine benefit.

**Table 5:** Side branch ballooning and protection techniques: rationale and considerations.

Technique	Principle	Main role	Key limitation
Side branch pre-dilatation	Dilate the side branch before main vessel stenting.	Facilitates stent delivery and expansion and side branch access in calcified or high-plaque-burden lesions.	Risk of plaque or carina shift and dissection; routine use may increase target-vessel failure.
Jailed wire technique	A guidewire is left in the side branch during main vessel stenting.	Preserves side branch access, marks the ostium, and modestly attenuates carina shift.	Limited protection against frank acute occlusion.
Jailed balloon technique	An uninflated or partly inflated balloon is positioned across the side branch ostium during stent deployment.	Allows immediate rescue of a high-risk side branch without re-crossing the stent.	Added procedural complexity; no demonstrated long-term clinical benefit.
Kissing balloon technique	Simultaneous inflation of main vessel and side branch balloons.	Optimizes the jailed side branch ostium and corrects main vessel malapposition.	Proximal over-stretch and stent deformation; should be used selectively.
Proximal optimization technique	A short non-compliant balloon expands the proximal stent to the carina.	Standard step that corrects proximal malapposition and facilitates side branch rewiring.	Does not fully reverse kissing-induced distortion; balloon position is critical.
Drug-coated balloon for the side branch	Delivers antiproliferative drug to the side branch without a permanent implant.	Treats a compromised side branch and limits late lumen loss with no added metal.	Variable drug transfer; requires lesion preparation; limited long-term data.

## 8 Limitations

Several limitations of this review should be acknowledged. First, this is a narrative rather than a systematic review; although the search and appraisal process is described in Section 1.4, studies were selected without a formal protocol, risk-of-bias assessment, or quantitative pooling, and some selection bias cannot be excluded. Second, the underlying evidence is markedly heterogeneous—in the definition of a significant side branch, side-branch size, treatment thresholds, ballooning techniques, endpoint definitions, and follow-up duration—which limits direct comparison across studies and precludes robust pooling. Third, much of the side-branch-specific evidence is observational or derives from subgroup or registry analyses that were not powered for side-branch outcomes, so several conclusions rest on lower-certainty data. Fourth, evidence generated in simple, non-left-main bifurcations cannot be extrapolated automatically to DEFINITION-complex lesions or to left-main disease, where the anatomy, myocardial territory at risk, and consequences of side-branch compromise differ substantially. Fifth, long-term, side-branch-specific outcomes—particularly ostial restenosis and durable branch patency—are sparsely reported, so the framework proposed here rests largely on short- and intermediate-term data and on mechanistic reasoning. These limitations temper the strength of the recommendations and reinforce the need for adequately powered, imaging-adjudicated randomized trials with standardized side-branch endpoints and multi-year follow-up.

## 9 Conclusions

Side branch ballooning is valuable but frequently overused. The evidence assembled here converges on a single conclusion: within the provisional stenting paradigm, SB ballooning should be selective rather than routine. Mechanistically, carina shift is a frequent and often dominant contributor to ostial compromise and is usually well managed without aggressive SB intervention; at the level of strategy, randomized trials show that a conservative SB approach did not worsen long-term outcomes relative to an aggressive one, and was associated with lower 3-year target-vessel failure, while reducing periprocedural injury, and that a stepwise provisional strategy remains the appropriate default for most bifurcations—including less-complex left-main disease, in which stepwise provisional and systematic two-stent treatment did not differ significantly (EBC MAIN)—whereas a planned two-stent strategy reduced target-lesion failure in DEFINITION-complex bifurcations and true left-main disease (DEFINITION II, DKCRUSH-V); technically, results are best optimized by routine proximal optimization and by the use of intracoronary imaging to refine procedural assessment, rewiring verification, sizing and optimization—with randomized trials supporting the clinical benefit of imaging-guided PCI in complex lesions, although neither OCTOBER nor RENOVATE-COMPLEX-PCI randomized rewiring or side-branch ballooning itself; and when the SB genuinely requires treatment, a drug-coated balloon is a promising, evidence-supported option that outperformed plain (uncoated) balloon angioplasty in appropriately selected compromised side branches. The unifying principle is to let physiology, imaging, and anatomy—not the angiographic appearance of a jailed ostium—decide whether and how to treat the side branch. The principal remaining evidence gap is the scarcity of adequately powered, imaging-adjudicated randomized trials with long-term, standardized SB-specific endpoints; closing this gap is the priority for placing SB ballooning on a firmer footing and refining its role in modern interventional cardiology.
